# The impact of armed conflict exposure on preterm birth, stillbirth, and neonatal anthropometric measures: a systematic review and meta-analysis

**DOI:** 10.3389/ijph.2026.1609784

**Published:** 2026-10-01

**Authors:** Amani N. Alansari, Alhan F. Sabbah, Amani Salim, Bejoy Varghese, Kalpana Singh, Alaa Ahmed Elshanbary

**Affiliations:** 1 Department of Pediatric Surgery, Hamad Medical Corporation, Doha, Qatar; 2 College of Education, Al-Aqsa University, Gaza, Palestine; 3 Faculty of Midwifery, Bethlehem University, Bethlehem, Palestine; 4 Department of Nursing Education, Hamad Medical Corporation, Doha, Qatar; 5 Nursing and Midwifery Research, Hamad Medical Corporation, Doha, Qatar; 6 Alexandria University Faculty of Medicine, Alexandria, Egypt

**Keywords:** armed conflict, birth weight, meta-analysis, neonatal outcomes, preterm birth

## Abstract

**Objectives:**

To assess the association between armed conflict exposure and neonatal outcomes, including preterm birth, stillbirth, neonatal mortality, and anthropometric measures.

**Methods:**

We conducted this systematic review following PRISMA guidelines. PubMed, EMBASE, Web of Science, and SCOPUS were searched from inception to January 2026. Studies examining neonatal outcomes among populations exposed to armed conflict, compared with non-exposed or pre-war populations were included. Meta-analyses were conducted using RevMan 5.4 software.

**Results:**

Seven studies encompassing diverse conflict settings met the inclusion criteria. No significant associations were observed for preterm birth (OR 0.80, 95% CI 0.61–1.05), stillbirth (OR 0.98, 95% CI 0.93–1.05), or neonatal mortality (OR 1.07, 95% CI 0.73–1.56) after accounting for heterogeneity. Conflict-exposed neonates had higher birth weight (MD 1.56 kg, 95% CI 0.55–2.56) and head circumference (MD 0.60 cm, 95% CI 0.47–0.74).

**Conclusion:**

The absence of adverse associations should not be interpreted as evidence of protection. These findings highlight methodological limitations in conflict epidemiology and the urgent need for community-based surveillance systems capturing outcomes among vulnerable populations unable to access healthcare during armed conflicts.

## Introduction

Neonatal health remains a critical global health priority, as deaths during the first 28 days of life account for a substantial proportion of under-five mortality worldwide [1]. In 2023, an estimated 17 neonatal deaths per 1,000 live births were reported globally, amounting to approximately 2.3 million neonatal deaths within the first month of life, with the highest rates in sub-Saharan Africa and South Asia [2]. Neonatal outcomes such as preterm birth, stillbirth, and neonatal mortality reflect not only biological vulnerability but also the performance of health systems and the social determinants of health [3]. Exposure to armed conflict may exacerbate risks through multiple pathways, including disruption of maternal health services, food insecurity, psychosocial stress, and displacement [4].

Armed conflict affects nearly every region of the world and continues to drive population displacement, structural violence, and healthcare collapse [4]. For example, prolonged conflicts in settings such as Syria, Yemen, South Sudan, Gaza, and Ukraine have been associated with deterioration in maternal and newborn health outcomes due to disintegration of health infrastructure and reduced access to skilled obstetric care [5, 6]. In conflict-affected zones, perinatal mortality has been reported at rates exceeding 40 stillbirths per 1,000 births and neonatal mortality above 50 per 1,000 live births, more than double global averages [7], although data quality remains variable due to weakened surveillance systems.

Armed conflict affects neonatal outcomes through multiple pathways. Maternal stress during conflict has been associated with preterm birth and LBW, while disruptions in antenatal care, emergency obstetric services, and transport increase the risk of delayed or inadequate management of complications [8, 9]. Food insecurity and malnutrition further impair fetal growth, contributing to neonatal morbidity and mortality [10]. Although prior reviews have explored maternal health in conflict settings [11, 12], quantitative synthesis of neonatal outcomes remains limited.

Studies examining neonatal outcomes in conflict settings are heterogeneous in design and scope, ranging from hospital-based cohorts to national registry trend analyses [13–15]. Some studies highlight increased prevalence of adverse outcomes such as LBW, preterm birth, and neonatal death among conflict-exposed populations [5, 16]. Conversely, other research underscores the complexity of associations, with certain regions demonstrating resilient outcomes despite ongoing conflict, possibly due to differential health system responses, population displacement toward care access points, or methodological variations in measurement [17, 18].

Understanding these effects is essential for prioritizing interventions and guiding resource allocation in humanitarian contexts. In this study, we conducted a systematic review and meta-analysis to assess the association between armed conflict exposure and neonatal outcomes, including preterm birth, stillbirth, neonatal mortality, and anthropometric measures.

## Methods

### Study design and reporting

This systematic review and meta-analysis was conducted in accordance with the Preferred Reporting Items for Systematic Reviews and Meta-Analyses (PRISMA) guidelines and Cochrane handbook [19, 20]. The study was registered in PROSPERO (CRD420261306923).

### Search strategy

A comprehensive literature search was performed in PubMed, EMBASE, Web of Science, and SCOPUS from database inception to January 2026. Search terms combined keywords and controlled vocabulary related to armed conflict (e.g., war, bombing, invasion, armed conflict) and neonates. The search strategy was adapted for each database. Reference lists of included studies were also screened to identify additional relevant articles Supplementary Table 1.

### Eligibility criteria

Studies were included if they examined populations exposed to armed conflict, incorporated a comparator group (such as pre-war versus wartime periods, exposed versus non-exposed groups, or temporal comparisons), reported neonatal outcomes, and used observational study designs including cohort, cross-sectional comparative, or registry-based approaches. Studies were excluded if they lacked a comparator group, were not related to war or armed conflict, were conference abstracts without sufficient data, or reported outcomes not relevant to neonatal health.

### Study selection and data extraction

All identified records were imported into reference management software and duplicates were removed. Two reviewers independently screened titles and abstracts for eligibility, followed by full-text assessment of potentially relevant studies. Disagreements were resolved through discussion until consensus was reached.

Data were extracted independently by two reviewers using a standardized data extraction form. Extracted information included study characteristics, population description, group sample sizes, comparator definition, and neonatal outcomes. Dichotomous outcomes were extracted as event counts and total sample sizes. Continuous outcomes were extracted as means and standard deviations. When outcomes were reported as rates per 1,000 births, event numbers were calculated using the reported denominators to enable meta-analysis.

### Quality assessment

Methodological quality was assessed independently by two reviewers. Cohort and historical cohort studies were evaluated using the Newcastle–Ottawa Scale [21], which assesses selection, comparability, and outcome domains with a maximum score of nine. Studies were categorized as high, moderate, or low quality based on established thresholds. The cross-sectional study was assessed using the NIH Quality Assessment Tool for Observational Cohort and Cross-Sectional Studies [22], which includes fourteen methodological domains and provides an overall rating of good, fair, or poor.

### Statistical analysis

Meta-analyses were conducted using Review Manager (RevMan 5.4). Dichotomous outcomes were pooled using odds ratios (ORs) with 95% confidence intervals (CIs) for preterm birth, stillbirth, and neonatal mortality, while continuous neonatal anthropometric outcomes, including birth weight, birth length, and head circumference, were pooled using mean differences (MDs) with 95% CIs. Statistical heterogeneity was evaluated using the Chi-square test and quantified with the I^2^ statistic. A fixed-effects model was initially applied; when substantial heterogeneity (P < 0.10) was detected, a random-effects model was used. Sensitivity analyses were performed by excluding studies contributing most to heterogeneity. Outcomes that could not be quantitatively pooled due to inconsistent definitions or reporting were synthesized narratively. No subgroup analysis was conducted. A p-value <0.05 was considered statistically significant.

## Results

A total of 7,902 records were identified through database searching. After removal of 1,885 duplicates, 6,017 records underwent title and abstract screening, of which 5,998 were excluded. Nineteen full-text reports were assessed for eligibility. Twelve reports were excluded due to lack of comparison (n = 6), not being war-related (n = 3), conference abstract (n = 2), or inappropriate outcomes (n = 1). Ultimately, seven studies met inclusion criteria and were included in both the systematic review and meta-analysis [13–15, 23–26] Figure 1.

**FIGURE 1:**
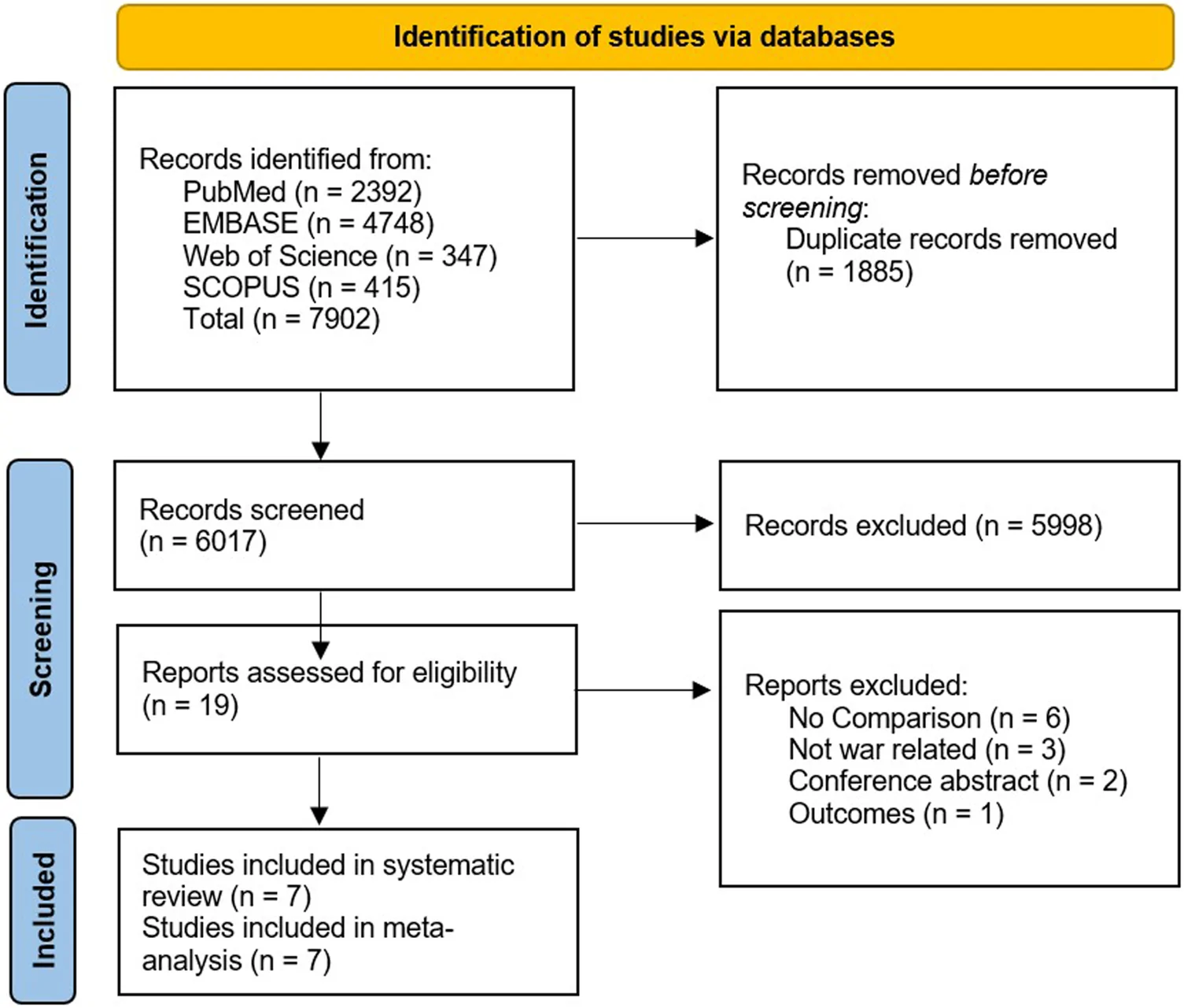
The preferred reporting items for systematic reviews and meta-analyses (PRISMA) flow diagram of study selection.

### Baseline and summary of the included studies

The studies were conducted in Ukraine, Poland, Serbia, Croatia/Bosnia, and Ethiopia, and employed retrospective cohort, historical cohort, cross-sectional comparative, and national registry designs. Sample sizes varied substantially, ranging from small hospital-based comparisons (n = 39) to national registry data exceeding 500,000 live births. Most studies compared pre-war and wartime periods, while one study contrasted residents with internally displaced persons. Baseline maternal characteristics were inconsistently reported. Table 1.

**TABLE 1:** Summary of the included studies.

ID	Country	Design	Study period	Setting	Population	Group sample sizes	Comparator	Baseline variables reported	Neonatal outcomes
Lakhno et al., [23]	Ukraine	Cross-sectional	Wartime (2022–2023)	Kharkiv hospital	Pregnant residents vs. internally displaced persons (IDPs)	Residents: 20 IDPs: 19	Resident vs. IDP status	Gestational age	Preterm birth, birth weight, length, head circumference, Apgar
Teka et al., [26]	Ethiopia (Tigray)	Retrospective comparative	2018–2019 vs. 2021–2022	Tertiary referral hospital	Women meeting WHO near-miss criteria	Pre-war: 691 Wartime: 428	Pre-war vs. wartime	Mode of delivery	Preterm birth, perinatal outcomes
Lakhno et al., [13]	Ukraine	Retrospective cohort	2021–2023	Kharkiv hospital	All deliveries across 3 years	2021: 2,914 2022: 956 2023: 1,288	Pre-war vs. war years	Not reported	Preterm birth
Vezhnovets et al., [15]	Ukraine	National registry trend	2012 vs. 2022	National birth registry	All registered live births	2012: 521,425 live births 2022: 199,619 live births	Pre-invasion vs. wartime year	Not reported (registry-level)	Neonatal mortality, early neonatal mortality, stillbirth
Liczbinska et al., [24]	Poland	Historical cohort	1930s–1940s (WWII)	Regional registry	Birth cohorts before vs. during WWII	Pre-WWII: 2,942 WWII: 4,116	Pre-WWII vs. WWII	Not reported	Stillbirth, neonatal death, birth weight
Maric et al., [14]	Serbia	Retrospective cohort	1996–1997, 1999–2000, 2003–2004	Single tertiary hospital (Belgrade)	Pregnant women delivering during bombing vs. non-bombing periods	Exposed: 1,198 Control: 2,617	Bombing exposure vs. non-exposed years	Maternal age (mean SD), parity, marital status, education, residence, multiple pregnancy, mode of delivery	Birth weight, preterm birth, stillbirth
Pavlinac et al., [25]	Croatia/Bosnia	Retrospective cohort	1988–1998	Split University hospital	Singleton births before, during, after war	Pre-war: 13,591 singleton War: 16,979 singleton	Pre-war vs. war	Maternal age, parity, delivery mode, fetal presentation	Preterm birth, stillbirth, early neonatal mortality, perinatal mortality

IDPs, internally displaced persons; WHO, world health organization; SD, standard deviation; WWII, second world war; PLTC, potentially life-threatening conditions; MNM, maternal near miss.

### Quality assessment

According to the Newcastle–Ottawa Scale assessment, cohort studies demonstrated moderate to high methodological quality, with scores ranging from 6 to 9 out of 9. Three studies were rated high quality (scores 7–9), reflecting strong selection procedures and objective outcome assessment, particularly in large registry and hospital-based cohorts [14, 15, 25]. Moderate-quality studies (6/9) primarily lacked adequate control for confounders, as they did not perform multivariable adjustment or matching for key maternal characteristics such as age, parity, socioeconomic status, and pre-existing medical conditions, thereby limiting comparability between exposed and unexposed groups [13, 24, 26]. Overall, the quality assessment indicated generally acceptable methodological quality, although important limitations were identified in confounding control and comparability Supplementary Table 2.

The cross-sectional study was rated as fair quality due to limited sample size, lack of confounder adjustment, and absence of sample size justification, despite clearly defined exposure and outcome measures [23]. These quality limitations were considered when interpreting the pooled findings. Supplementary Table 3.

### Outcomes

#### Preterm birth

Preterm birth showed no significant association with conflict exposure in the fixed-effects model based on the pooled analysis of 4 studies (OR 0.96, 95% CI 0.83–1.10; p = 0.54), with substantial heterogeneity (I^2^ = 85%, p < 0.001). After resolving heterogeneity using a random-effects model and excluding Teka et al. 2025, variability decreased to moderate levels (I^2^ = 46%, p = 0.16), and the pooled estimate remained non-significant (OR 0.80, 95% CI 0.61–1.05; p = 0.11) Figures 2A,B.

**FIGURE 2:**
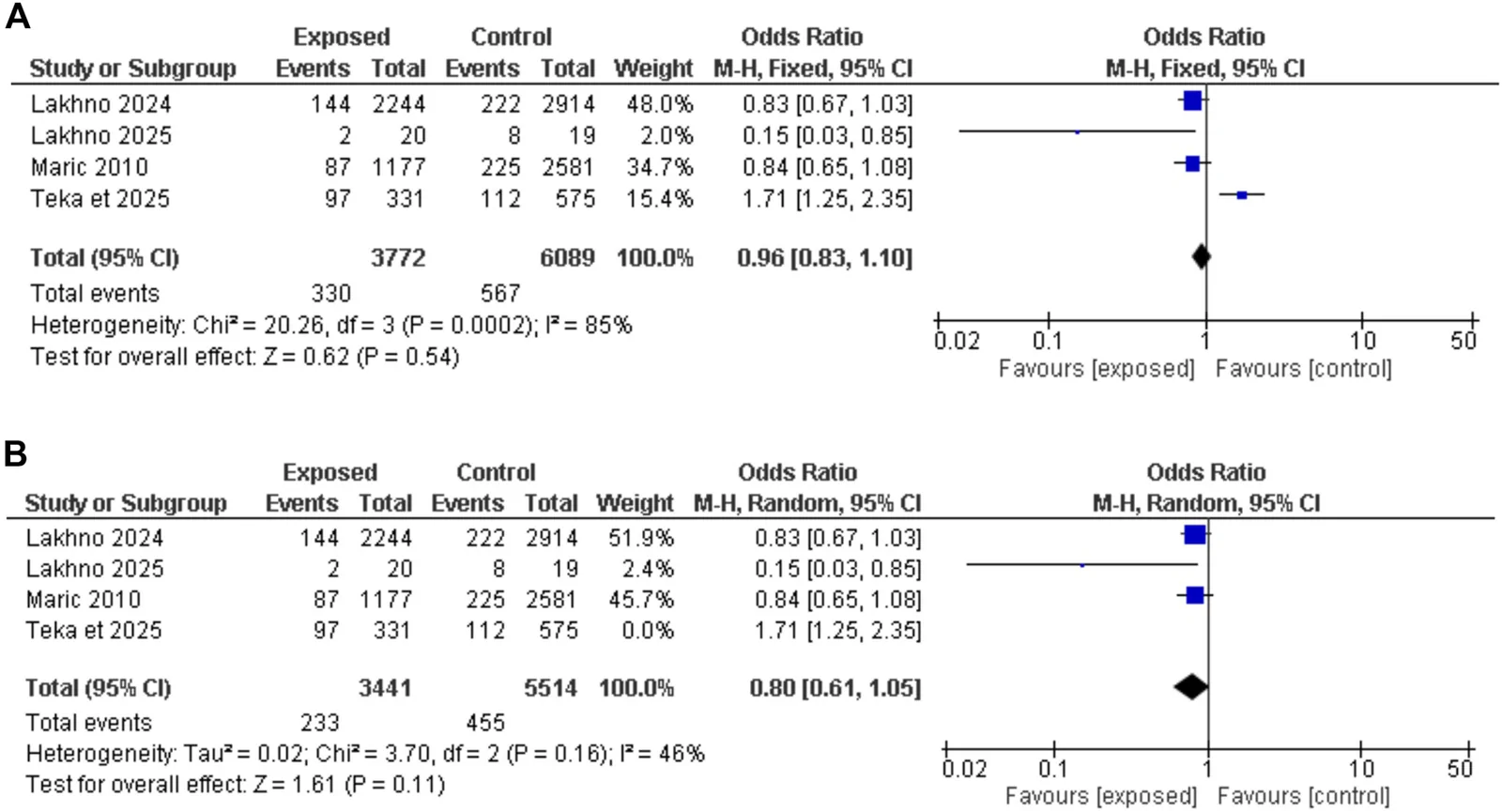
Forest Plot of Preterm Birth. **(A)** Fixed-effect model before addressing substantial heterogeneity; **(B)** random-effects model after excluding the study contributing most to heterogeneity. The comparison was between conflict-exposed and non-exposed/pre-war populations.

#### Stillbirth

For stillbirth, the pooled fixed-effects model of 4 studies demonstrated no significant difference between exposed and control groups (OR 1.00, 95% CI 0.94–1.07; p = 0.94), although considerable heterogeneity was present (I^2^ = 80%, p = 0.002), Figure 3A. After applying a random-effects model and excluding Liczbinska 2020, heterogeneity was eliminated (I^2^ = 0%, p = 0.42), and the pooled estimate remained non-significant (OR 0.98, 95% CI 0.93–1.05; p = 0.63) Figure 3B.

**FIGURE 3:**
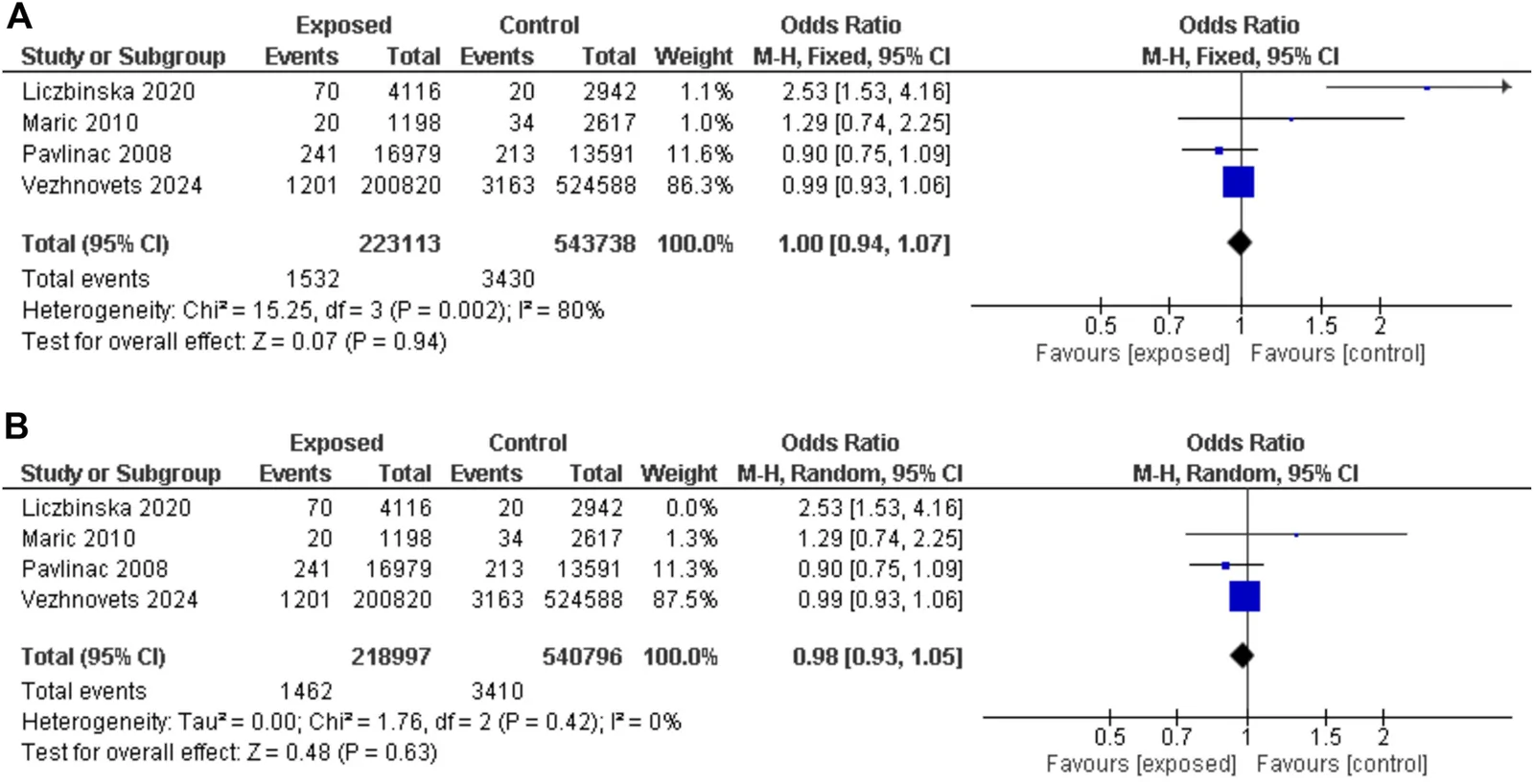
Forest Plot of Stillbirth. **(A)** Fixed-effect model before addressing substantial heterogeneity; **(B)** random-effects model after excluding the study contributing most to heterogeneity. The comparison was between conflict-exposed and non-exposed/pre-war populations.

#### Neonatal death

For neonatal death, the pooled fixed-effects model of 4 studies showed a significant reduction among exposed populations (OR 0.78, 95% CI 0.71–0.85; p < 0.00001); however, heterogeneity was considerable (I^2^ = 89%, p < 0.00001). After applying a random-effects model, heterogeneity remained high (I^2^ = 89%). The pooled estimate became non-significant (OR 1.07, 95% CI 0.73–1.56; p = 0.74) Figures 4A,B.

**FIGURE 4:**
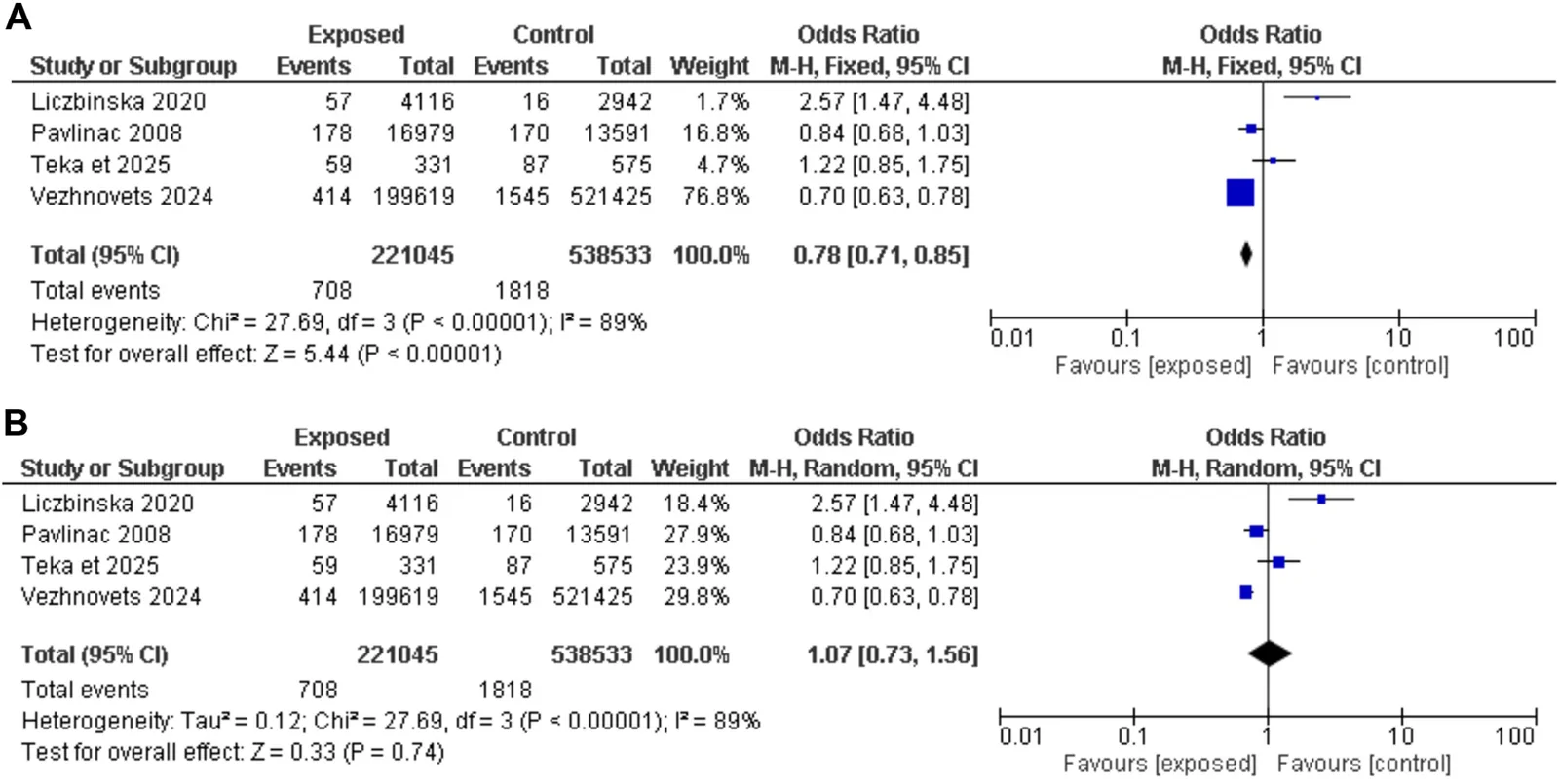
Forest Plot of Neonatal Death. **(A)** Fixed-effect model; **(B)** random-effects model. The comparison was between conflict-exposed and non-exposed/pre-war populations.

#### Birth weight (kg), birth length (cm), and head circumference (cm)

For birth weight (BW), the random-effects model of two studies demonstrated a statistically significant increase among exposed neonates (MD 1.56, 95% CI 0.55 to 2.56; p = 0.002), although heterogeneity was considerable (I^2^ = 92%, p = 0.0005). In contrast, birth length showed no significant difference between groups (MD 3.18, 95% CI −3.79 to 10.16; p = 0.37), based on the pooled analysis of two studies, with similarly high heterogeneity (I^2^ = 89%, p = 0.003) Figure 5.

**FIGURE 5:**
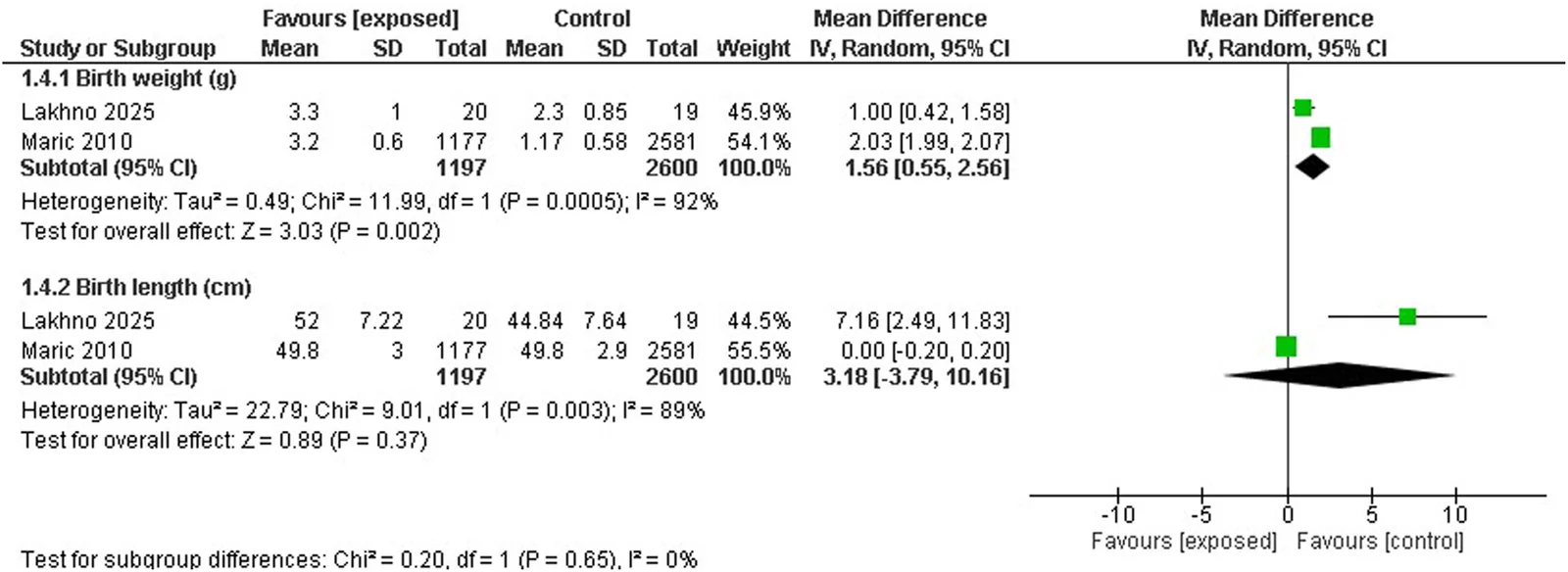
Forest Plot of Neonatal Anthropometric Measures. Birth weight; birth length. The comparison was between conflict-exposed and non-exposed/pre-war populations.

Also, head circumference demonstrated a significant increase among exposed neonates (MD 0.60, 95% CI 0.47 to 0.74; p < 0.00001), with low-to-moderate heterogeneity (I^2^ = 35%). Supplementary Figure 1

### Qualitative synthesis

Beyond the pooled outcomes, several neonatal indicators were reported but not meta-analyzed due to inconsistent definitions or limited reporting. Lakhno et al. [23] reported significantly shorter birth length (44.84 ± 7.64 cm vs. 52.0 ± 7.22 cm) and lower Apgar scores (6.74 ± 2.05 vs. 7.7 ± 2.08) among internally displaced infants compared with residents [23]. Lakhno et al. [13] documented variations in gestational age (GA) distribution across 2021–2023 without detailed anthropometric stratification [13]. Liczbinska et al. [24] additionally reported changes in miscarriage rates (895 pre-war vs. 851 during WWII) [24].

## Discussion

### Summary of main findings

Our systematic review and meta-analysis of seven studies across diverse geographic settings found no significant associations between armed conflict exposure and preterm birth, stillbirth, or neonatal mortality after addressing heterogeneity. Unexpectedly, exposed neonates showed significantly higher BW and head circumference than non-exposed neonates.

These counterintuitive findings warrant careful interpretation. Several mechanisms may explain the paradoxical increase in anthropometric measures. First, survival bias likely plays a substantial role, infants who survive to delivery in conflict settings may represent a more reliable subset of pregnancies, as those with compromised fetal health may be more likely to result in early pregnancy loss or SB that goes unreported in conflict zones with weakened surveillance systems [7, 27]. Second, population displacement patterns may concentrate healthier, more socioeconomically advantaged families near functioning health facilities, while those unable to relocate face worse outcomes that remain invisible to hospital-based studies [17, 28]. Third, the included studies demonstrated considerable methodological heterogeneity in exposure definition, timing assessment, and comparison group selection, which may affect true associations.

The absence of significant findings for SB and neonatal mortality contradicts both theoretical expectations and prior observational evidence suggesting heightened perinatal risks in conflict settings [4, 17]. However, this lack of association may reflect the limitations of available data rather than true biological protection. Most included studies relied on facility-based data, potentially missing the most vulnerable populations who cannot access healthcare. Finally, the moderate quality of several studies, particularly the absence of multivariable adjustment for critical confounders such as maternal socioeconomic status and nutritional status, limits our ability to isolate the independent effects of conflict exposure [13, 24, 26].

The included studies had strengths such as large registry or hospital-based datasets, defined comparison groups, and objective outcome measures. However, several were retrospective, single-center, or historical studies, with potential selection bias, temporal confounding, and limited adjustment for maternal and socioeconomic factors. These differences should be considered when interpreting the pooled estimates.

### Comparison with previous literature

Our findings diverge substantially from prior research examining armed conflict and neonatal outcomes. A comprehensive study by Le and Nguyen analyzing 53 developing countries over three decades found that intrauterine exposure to armed conflict reduced BW by 2.8% and increased LBW incidence by 3.2 percentage points [29]. Similarly, Mansour and Rees, investigating the al-Aqsa Intifada’s impact, documented modest increases in LBW risk among conflict-exposed Palestinian infants [30]. Studies, utilizing large population-based datasets and difference-in-differences designs likely captured more representative samples than the predominantly hospital-based studies in our review.

A systematic review by Keasley et al. [31] identified evidence for increased LBW risk across nine studies of mothers exposed to armed conflicts in diverse settings including Libya, Bosnia, Palestine, and Afghanistan [31]. Their narrative synthesis highlighted consistent patterns of adverse pregnancy outcomes, though methodological heterogeneity limited quantitative pooling of all outcomes. Importantly, that review encompassed studies examining LBW as a binary outcome rather than continuous BW measures, which may explain some discordance with our findings.

More recently, Jawad et al. [11] conducted a regression analysis of 181 countries from 2000 to 2019, demonstrating that conflicts classified as wars were associated with increased maternal and infant mortality, though notably, they found no evidence of association with neonatal mortality, a finding consistent with our results [11]. This suggests that conflict’s impact on neonatal survival may be more nuanced and context-dependent than its effects on broader maternal and child health indicators.

A meta-analysis by Behboudi-Gandevani et al. [17] examining immigrants from conflict zones found increased odds of small for GA and neonatal mortality compared to host populations, highlighting that conflict effects may extend beyond the immediate war zone through displacement and refugee experiences [17].

### Interpretation and clinical implications

The clinical implications of our findings must be considered cautiously given the methodological limitations and potential for selection bias. While the absence of significant adverse associations may offer superficial reassurance, this likely reflects data gaps rather than genuine protection. Healthcare providers caring for conflict-affected populations should maintain heightened awareness of adverse outcomes regardless of these pooled estimates.

The paradoxical anthropometric increases highlight that hospital-based datasets systematically undercount the most vulnerable, underscoring the need for community-based surveillance and mobile health initiatives [6, 18]. Humanitarian organizations must prioritize establishing parallel data collection mechanisms outside hospital settings to capture the full spectrum of outcomes.

Beyond immediate obstetric outcomes, the health effects of war may also be mediated by exposure to hazardous munitions and toxic conflict remnants. Reported use of incendiary substances such as white phosphorus in populated areas has raised serious humanitarian and public health concerns, particularly regarding possible long-term reproductive, developmental, and intergenerational consequences [32]. These risks are rarely measurable in routine hospital-based datasets, reinforcing the likelihood that current pooled estimates underestimate the true burden of conflict on maternal and neonatal health.

From a public health perspective, these findings highlight the critical importance of maintaining essential maternal and newborn care services during armed conflicts. Even if hospital-based outcomes appear preserved, this may reflect successful triage and resource allocation rather than absence of need. Interventions should focus on ensuring access to midwifes, emergency obstetric care, and nutritional support for all pregnant women in conflict zones, particularly those unable to reach formal healthcare facilities [12].

### Strengths and limitations

This systematic review and meta-analysis represents, to our knowledge, the first quantitative synthesis examining the association between armed conflict exposure and neonatal outcomes. Studies from diverse geographic regions were included, enhancing the relevance and applicability of findings across different conflict settings. Methodological quality was systematically evaluated using validated assessment tools, allowing transparent appraisal of study strengths and limitations.

However, several important limitations constrain interpretation. First, the small number of included studies (n = 7) and their predominant reliance on hospital-based data limit representativeness. Second, Substantial statistical heterogeneity across outcomes reflects methodological diversity in exposure definitions, comparison groups, and confounder adjustment. Third, most studies demonstrated moderate quality with inadequate control for socioeconomic and nutritional confounders that may independently affect neonatal outcomes. Fourth the absence of individual patient data precluded subgroup analyses by conflict intensity, exposure timing, or maternal characteristics. Fifth, publication bias assessment was not feasible given the limited number of studies. Sixth, many studies do not fully reflect the context of long-standing conflicts in the region (e.g., Syria, Libya, Palestine, Lebanon, Iraq, Iran, Sudan, and Yemen). Seventh, maternal and neonatal outcomes in these settings are influenced not only by conflict but also by economic collapse, displacement, food insecurity, and disruption of healthcare systems, which may produce large-scale ramifications that are difficult to disentangle from the direct effects of war. Finally, the ecological nature of conflict exposure measurement in many studies prevents definitive causal inference about individual-level effects.

### Conclusions and recommendations

We observed no significant associations between conflict exposure and preterm birth, stillbirth, or neonatal mortality, alongside paradoxically increased birth weight and head circumference. Rather than biological protection, this pattern likely indicates facility-level selection bias, missed out-of-hospital deliveries, and fragile surveillance in war zones. Therefore, the apparent lack of adverse associations likely reflects data limitations rather than the absence of serious conflict-related effects. Future research requires community-based prospective cohorts with multivariable adjustment (nutrition, socioeconomic indicators, healthcare access) and exposure timing. Clinicians should maintain heightened risk awareness, while policymakers and humanitarians should enforce health-facility protections and invest in community-level outreach and parallel surveillance to reach the most vulnerable pregnant populations. Ultimately, preventing armed conflict and spreading peace remains the most effective intervention for protecting maternal and neonatal health globally.
