# Supplementary material for: The impact of armed conflict exposure on preterm birth, stillbirth, and neonatal anthropometric measures: a systematic review and meta-analysis
Source: Int J Public Health. 2026 Oct 1;71:1609784. doi: 10.3389/ijph.2026.1609784 (PMC13668762; doi:10.3389/ijph.2026.1609784)
Supplement: Supplementary file 1 [file Supplementaryfile1.docx]

**The Impact of Armed Conflict Exposure on Preterm Birth, Stillbirth, and Neonatal Anthropometric Measures: A Systematic Review and Meta-Analysis**

**Journal name:** The International Journal of Public Health

**Supplementary Tables Legend:**

**Table S1:** Advanced search strategy for each database

**Table S2:** Quality assessment for the included cohort studies

**Table S3:** Quality assessment for the included cross-sectional study

**Supplementary Figures Legend:**

**Figure S1:** Forest Plot of Head Circumference

**Table S1.** Advanced search strategy for each database.

| **Database** | **Advanced Search Strategy** |
| --- | --- |
| **PubMed** | ("War"[MeSH] OR "Armed Conflicts"[MeSH] OR "Violence"[MeSH] OR war[tiab] OR "armed conflict*"[tiab] OR warfare[tiab] OR "military conflict*"[tiab] OR "political violence"[tiab] OR bombing*[tiab]) AND ("Pregnancy"[MeSH] OR "Pregnant Women"[MeSH] OR "Maternal Exposure"[MeSH] OR pregnan*[tiab] OR pregnancy[tiab] OR maternal*[tiab] OR "antenatal exposure"[tiab] OR "prenatal exposure"[tiab]) AND ("Infant, Newborn"[MeSH] OR "Perinatal Mortality"[MeSH] OR "Infant Mortality"[MeSH] OR "Birth Weight"[MeSH] OR neonat*[tiab] OR newborn*[tiab] OR perinatal[tiab] OR "low birth weight"[tiab] OR preterm[tiab]) |
| **Scopus** | TITLE-ABS ((war OR "armed conflict*" OR warfare OR "military conflict*" OR "political violence" OR bombing*) AND (pregnan* OR pregnancy OR maternal* OR "antenatal exposure" OR "prenatal exposure") AND (neonat* OR newborn* OR perinatal OR "low birth weight" OR preterm OR "infant mortality" OR "perinatal mortality")) |
| **Web of Science (WoS)** | TS=((war OR "armed conflict*" OR warfare OR "military conflict*" OR "political violence" OR bombing*) AND (pregnan* OR pregnancy OR maternal* OR "antenatal exposure" OR "prenatal exposure") AND (neonat* OR newborn* OR perinatal OR "low birth weight" OR preterm OR "infant mortality" OR "perinatal mortality")) |
| **Embase** | ('war'/exp OR 'armed conflict'/exp OR 'violence'/exp OR war:ti,ab OR 'armed conflict*':ti,ab OR warfare:ti,ab OR 'political violence':ti,ab OR bombing*:ti,ab) AND ('pregnancy'/exp OR 'pregnant woman'/exp OR 'maternal exposure'/exp OR pregnan*:ti,ab OR pregnancy:ti,ab OR maternal*:ti,ab OR 'antenatal exposure':ti,ab OR 'prenatal exposure':ti,ab) AND ('newborn'/exp OR 'perinatal mortality'/exp OR 'infant mortality'/exp OR 'birth weight'/exp OR neonat*:ti,ab OR newborn*:ti,ab OR perinatal:ti,ab OR 'low birth weight':ti,ab OR preterm:ti,ab) |

**Table S2.** Quality assessment for the included cohort studies.

| **ID** | **Selection (4)** | **Comparability (2)** | **Outcome (3)** | **Total Score (9)** | **Quality Level** |
| --- | --- | --- | --- | --- | --- |
| **Teka 2025** | ★★★ | ★ | ★★ | 6/9 | Moderate |
| **Lakhno 2024** | ★★★ | ★ | ★★ | 6/9 | Moderate |
| **Vezhnovets 2024** | ★★★★ | ★ | ★★ | 7/9 | High |
| **Liczbinska 2020** | ★★★ | ★ | ★★ | 6/9 | Moderate |
| **Maric 2010** | ★★★★ | ★★ | ★★★ | 9/9 | High |
| **Pavlinac 2008** | ★★★★ | ★ | ★★★ | 9/9 | High |

**Table S3.** Quality assessment for the included cross-sectional study.

| **Domain** | **Lakhno 2025** |
| --- | --- |
| 1. Research Question Clearly Stated | Yes |
| 2. Study Population Clearly Defined | Yes |
| 3. Participation Rate ≥50% | NR |
| 4. Subjects from Similar Population | Yes |
| 5. Sample Size Justified | No |
| 6. Exposure Measured Before Outcome | NA |
| 7. Timeframe Sufficient | Yes |
| 8. Exposure Levels Clearly Defined | Yes |
| 9. Exposure Measured Reliably | Yes |
| 10. Exposure Assessed More Than Once | NA |
| 11. Outcomes Clearly Defined & Reliable | Yes |
| 12. Outcome Assessors Blinded | NR |
| 13. Loss to Follow-up ≤20% | NA |
| 14. Confounders Measured & Adjusted | No |
| **Overall Rating** | **Fair** |

**Figure S1.** Forest Plot of Head Circumference
